# Equilibrium Optimization Algorithm with Ensemble Learning Based Cervical Precancerous Lesion Classification Model

**DOI:** 10.3390/healthcare11010055

**Published:** 2022-12-25

**Authors:** Rasha A. Mansouri, Mahmoud Ragab

**Affiliations:** 1 Department of Biochemistry, Faculty of Sciences, King Abdulaziz University, Jeddah 21589, Saudi Arabia; 2 Information Technology Department, Faculty of Computing and Information Technology, King Abdulaziz University, Jeddah 21589, Saudi Arabia; 3 Department of Mathematics, Faculty of Science, Al-Azhar University, Naser City, Cairo 11884, Egypt

**Keywords:** medical imaging, healthcare, decision making, cervical cancer, ensemble learning

## Abstract

Recently, artificial intelligence (AI) with deep learning (DL) and machine learning (ML) has been extensively used to automate labor-intensive and time-consuming work and to help in prognosis and diagnosis. AI’s role in biomedical and biological imaging is an emerging field of research and reveals future trends. Cervical cell (CCL) classification is crucial in screening cervical cancer (CC) at an earlier stage. Unlike the traditional classification method, which depends on hand-engineered or crafted features, convolution neural network (CNN) usually categorizes CCLs through learned features. Moreover, the latent correlation of images might be disregarded in CNN feature learning and thereby influence the representative capability of the CNN feature. This study develops an equilibrium optimizer with ensemble learning-based cervical precancerous lesion classification on colposcopy images (EOEL-PCLCCI) technique. The presented EOEL-PCLCCI technique mainly focuses on identifying and classifying cervical cancer on colposcopy images. In the presented EOEL-PCLCCI technique, the DenseNet-264 architecture is used for the feature extractor, and the EO algorithm is applied as a hyperparameter optimizer. An ensemble of weighted voting classifications, namely long short-term memory (LSTM) and gated recurrent unit (GRU), is used for the classification process. A widespread simulation analysis is performed on a benchmark dataset to depict the superior performance of the EOEL-PCLCCI approach, and the results demonstrated the betterment of the EOEL-PCLCCI algorithm over other DL models.

## 1. Introduction

Cervical cancer (CC) ranks as the fourth most common cancer in females. As per the statistical report by WHO, approximately 604,000 new cases occurred worldwide in 2020, particularly 6.5% of cancer cases in females [1]. Although the initial treatment rate of CC is high, lack of symptoms and signs hinders the initial diagnoses. An effective screening program may prevent CC deaths and decrease the persistence and incidence of the disease. The statistical reports stated that over 311,000 CC deaths occurred annually [2]. Because of amateur healthcare staff and inadequate screening funds, CC screening facilities seem to be very scarce in developing nations [3]. Thus, employing effective and automated screening techniques is essential to reduce the cost of initial detection of CC. CC screening follows the following workflow: colposcopy, HPV test, biopsy, and PAP smear test or cytology.

Numerous tools reinforce the task, which make it inexpensive, practical, and very effective [4]. The PAP smear image screening can be used for the treatment of CC; however, it needs several microscopic analyses to find non-cancer and cancer patients, and even if it takes more time and necessitates skilled professionals, there comes a chance of missing the positive case with the use of the traditional screening technique [5]. The HPV testing and PAP smear are expensive medications and offer less sensitivity. In contrast, colposcopy treatment can be broadly employed in developing nations. Colposcopy screening is employed to address the limitations of HPV testing and PAP smear images [6]. The cervical and other cancers are probably treated at the initial level. However, the lack of symptoms at this phase will hinder the initial diagnosis. CC deaths are evaded by effective screening methods and result in impermanence and lowered sickness [7]. CC screening facilities are very sparse in middle-and-low-income countries due to a lack of educated and experienced healthcare professionals and inadequate funding to fund screening mechanisms.

Some of the important advancements of deep learning (DL) in various applications are battery health monitoring, natural language processing (NLP), forecasting, and computer vision (CV) [8]. Medical image processing, which includes registration, classification, segmentation, and identification, had a significant role in diagnosing disease. Medical images of blood smears, MRI, ultrasound, and CT constitute the major part of the image data processed [9]. The multilayer neural network perception system of DL has more extracted features in images and was anticipated to overcome the challenges plaguing standard CAD systems. Still, the DL methods have to be reinforced with a wide range of datasets, particularly for positive cases [10]. Several ensemble learning and transfer learning (TL) methods were used to solve this problem [11,12,13].

This study develops an equilibrium optimizer with ensemble learning-based cervical precancerous lesion classification on colposcopy images (EOEL-PCLCCI) technique. The presented EOEL-PCLCCI technique mainly focuses on identifying and classifying cervical cancer on colposcopy images. In the presented EOEL-PCLCCI technique, the DenseNet-264 architecture is used for the feature extractor. Since the trial and error method for hyperparameter tuning is tedious and erroneous, metaheuristic algorithms can be applied. Therefore, in this work, we employ the EO algorithm for the parameter selection of the DenseNet model. An ensemble of weighted voting classifiers, namely long short-term memory (LSTM) and gated recurrent unit (GRU), is used for the classification process. A widespread simulation analysis is performed on a benchmark dataset to depict the enhanced performance of the EOEL-PCLCCI algorithm.

## 2. Related Works

Khamparia et al. [14] developed a new Internet of Health Things (IoHT)-based DL algorithm for classifying and recognizing CC in pap smear images with a TL model. Then, CNN was fused with outdated ML approaches. In this work, feature extraction from cervical images can be carried out by pre-trained CNN modules such as ResNet50, InceptionV3, VGG19, and SqueezeNet and are fed into flattened and dense layers for the classification of normal and abnormal CCLs. Shi et al. [15] recommend a classification of CCLs based GCN model. The study aims at exploring the possible relations of CCL images for enhancing the accuracy of classification. The CNN feature of each CCL image was clustered initially, and the inherent relationship of images can be exposed earlier through the clustering. A graph model has been constructed to capture the fundamental correlation among the clusters further.

Allehaibi et al. [16] propose a CCL segmentation with mask regional CNN (Mask R-CNN) and categorizes by a small VGG-like Net. ResNet10 uses prior knowledge and spatial information as the backbone of Mask R-CNN. Chen et al. [17] developed a TL-based snapshot ensemble (TLSE) technique by incorporating them in a unified and coordinated manner. SE technique offers ensemble advantages within a single model training method, whereas TL emphasizes the smaller sampling problems in CCL classification. Archana and Panicker [18] advise a new methodology for the multiclass classification of CCLs with less computing power, optimum feature extraction, and minimal parameters. The application of ConvNet with the TL method validates substantial diagnoses of cancer cells. 

Dong et al. [19] proposed a cell classification technique which combines artificial and Inceptionv3 features that considerably enhance the performance of CCL detection. Furthermore, the study inherits the stronger learning capability from TL to address the under-fitting problems and perform effectual DL training with a less quantity of medicinal datasets and accomplishes precise and effective CCL image classification based on Herlev data. Li et al. [20] introduced an L-PCNN which incorporates a global context dataset and attention module for categorizing CCLs. The cell image was transferred to the improved ResNet50 model for extracting DL features. For extracting deep features, every convolutional block presents an attention module for guiding the network to emphasize the cell region. Next, the network includes a pyramid pooling layer and an LSTM for aggregating image features in distinct areas.

## 3. The Proposed Model

**Figure 1:**
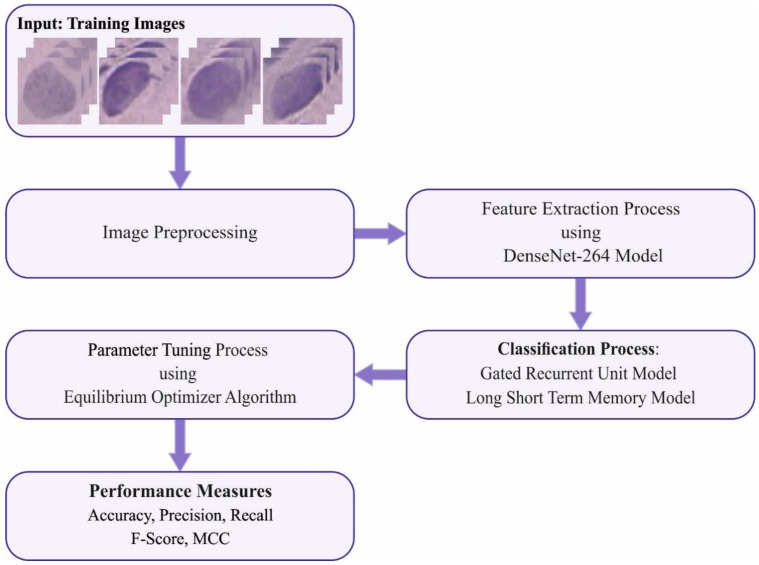
Working process of EOEL-PCLCCI system.

In this study, we introduced an automated cervical cancer classification model, the EOEL-PCLCCI technique, on colposcopy images. The EOEL-PCLCCI technique uses a DenseNet-264 feature extractor, EO hyperparameter optimizer, and weighted voting classifier. Figure 1 illustrates the working process of the EOEL-PCLCCI system.

### 3.1. Feature Extraction

In the presented EOEL-PCLCCI technique, the DenseNet-264 architecture is used for the feature extraction. In the typical CNN, every layer is gradually interconnected, making the network difficult to go deeper and wider. Meanwhile, it has a gradient exploding or vanishing problem [21]. Consequently, DenseNet analyzes the module by successively concatenating all the feature maps instead of outputting feature maps from every prior layer in the following:

(1) $$x_{l}=H_{l}\left(x_{l-1}\right)\;$$

(2) $$x_{l}=H_{l}\left(x_{l-1}\right)+x_{l-1}$$

(3) $$x_{l}=H_{l}\left(\left[x_{0},x_{1},\;x_{2},\;\ldots ,x_{l-1}\right]\right)\;$$

H indicates the nonlinear function from the expression, and  l characterizes the layer index. $x_{l}$ symbolizes the feature of l-th layers. DenseNet concurs all the feature maps from previous layers, indicating that all the feature maps are propagated toward the last layer and interconnected toward the new feature maps. The recently designed DenseNet has certain benefits, namely feature reutilization and reduction in gradient exploding or vanishing problems. Once the size of feature maps continuously changes, the concatenation function becomes impossible to be implemented. Among the dense blocks, transition layers exist: convolution, pooling, and BN operations. Meanwhile, each layer receives feature maps from all the previous layers. Note that k feature maps are constructed for each $\;H_{l}$ operation. Meanwhile, there exist five layers, and we obtain $k_{0}+4k$ feature maps. $k_{0}$ symbolizes the number of feature maps from prior layers.

However, there exists a huge quantity of inputs, and bottleneck layers are introduced for the DenseNet, viz., implemented using the 1×1 convolution layer beforehand 3×3 convolution layers that are beneficial to save the computational cost and decrease the feature map. Subsequently, considering the model compactness, a transition layer is applied to reduce the feature maps: consider m feature maps are constructed using DenseBlock and assume the compression factors $\;\theta \epsilon \left(0,1\right)$. If θ=1, the quantity of feature maps remains unchanged. The DenseNet module encompasses transition layers, input layers, Dense Blocks, and global average pooling (GAP). The transition layer comprises the BN layer, 1×12×2 convolution, and average pooling layers with a stride of 2.

To adjust the hyperparameters associated with the DenseNet-264 model, the EO algorithm is exploited in this work. The fundamental idea of single objective EO has been established based on the dynamic mass balance [22]. This characteristic can maintain the balance between exploitation and detection and the ability to retain flexibility among individual solutions. In the initialization, EO uses a certain group, while each particle explains the vector of focus that has solutions to the problem.

(4) $$Y_{j}^{initial}=lb+rand_{j}\left(ub-lb\right),\;j=0,1,2,3,\ldots ,n\;\;\;\;$$

$Y_{j}^{initial}$ denotes the vector focus on *j*th particles, ub and lb represent the upper and lower boundaries of each parameter, $rand_{j}$ indicates the arbitrary integer within [0, 1], and n shows the number of particles. Hence, it assigns an equilibrium candidate to the optimal four particles from the population. In the exploitation and exploration methods, these five equilibrium candidate assists EO. The initial four candidates seek optimal exploration. However, the 5th candidate with average values seeks alteration from exploitation.

(5) $${\vec{C}}_{\mathrm{eq},\mathrm{pool}}=\left\{{\vec{C}}_{\mathrm{eq}\left(1\right)}\;,{\vec{C}}_{\mathrm{eq}\left(2\right)},{\vec{C}}_{\mathrm{eq}\left(3\right)},{\vec{C}}_{\mathrm{eq}\left(4\right)},{\vec{C}}_{\mathrm{eq}\left(\mathrm{ave}\right)}\right\}$$

The upgrade of concentration enables EO to balance exploitation and exploration equally:

(6) $$\vec{F}=e^{-\vec{\lambda }\left(t-t_{0}\right)}$$

Equation (6) $\;\vec{\lambda }$ indicates the arbitrary integer within [0, 1], and t reduces as the iteration amount enhances.

(7) $$t={\left(1-\frac{It}{\mathrm{Max}\_it}\right)}^{\left(a_{2}\frac{It}{\mathrm{Max}\_it}\right)}\;\;\;$$

It and Max_it denote existing and maximal iteration counts, and $a_{2}$ shows the constant control of the ability for exploiting. Another parameter, $a_{1}$, has been employed to enhance exploration and exploitation:

(8) $$t=\frac{1}{\vec{\lambda }}\ln\left(-a_{1}\;\mathrm{sign}\;\left(\vec{r}-0.5\right)\left[1-e^{\vec{-\vec{\lambda }t}}\right]\right)+t$$

The generation rate is denoted as G rises exploitation:

(9) $$\vec{G}={\vec{G}}_{0}e^{-\vec{l}\left(t-t_{0}\right)}$$

Equation (9) $\vec{l}$ denotes the arbitrary number within [0, 1], and the initial generation rate represented by ${\vec{G}}_{0}$:

(10) $${\vec{G}}_{0}=G\vec{C}P\left({\vec{C}}_{\mathrm{eq}}-\vec{\lambda }\vec{C}\right)\;\;$$

(11) $$G\vec{C}P=\left\{\begin{array}{l} 0.5r_{1},r_{2}\geq GP\\ 0,\;r_{2}<GP \end{array}\right.$$

From the expression, the arbitrary integers are denoted by $r_{1}$ and $r_{2}$ and vary between zero and one. The vector $\vec{GC}P$ represents the variable which controls the generation rate executed for the upgrading phase.

(12) $$\vec{C}=\vec{C}+\left(\vec{C}-{\vec{C}}_{\mathrm{eq}}\right)\;\cdot \vec{F}+\frac{\vec{C}}{\vec{\lambda }V}\left(1-\vec{F}\right)\;$$

The value of V corresponds to 1. 

### 3.2. Weighted Voting-Based Ensemble Classification

An ensemble of weighted voting classifiers, GRU and LSTM, is used for the classification process. The DL algorithm is incorporated, and the maximal result is preferred by the weighted voting method [23]. Considering the D base classification and amount of classes as n for voting, the predictive class $c_{k}$ of weighted voting for every instance, k, can be defined by:

(13) $$c_{k}=\arg\underset{j}{\max}\overset{D}{\underset{i=1}{\sum }}\left(\Delta_{ji}\times w_{i}\right)\;\;$$

The expression $\Delta_{\mathrm{ji}}$ indicates the binary variable. As soon as the i-th base classification classifies the k instances into j-th classes, then $\Delta_{\mathrm{ji}}=1$, or else, $\Delta_{\mathrm{ji}}=0$. $w_{i}$ shows the weight of i-th base classifications:

(14) $$Acc=\frac{\sum_{k}\left\{1|c_{k}\;\mathrm{is}\;\mathrm{the}\;\mathrm{true}\;\mathrm{class}\;\mathrm{of}\;\mathrm{instance}\;k\right\}}{\mathrm{Size}\;\mathrm{of}\;\mathrm{test}\;\mathrm{instances}}\times 100\%.\;\;$$

#### 3.2.1. GRU Model

GRU is an LSTM network which inherits the advantages of RNN: it learns features automatically and effectively models long dependency datasets. It is utilized for short-term traffic prediction. Intuitively, input and forget gates are integrated as a reset gate in GRU, which determines how to incorporate the novel input dataset in the previous time. Another gate in GRU is an update gate; it determines the information stored from the previous time to the existing time. Therefore, GRU is one gate lower than LSTM. This makes the GRU network have faster training speed and lesser variables and needs lesser datasets for efficiently generalizing the system:

(15) $$z_{n}=\sigma ƒ\left(W_{z}\cdot \left[h_{n-1},\;x_{n}\right]\right)\;\;$$

(16) $$r_{n}=\sigma ƒ\left(W_{r}\cdot \left[h_{n-1},\;x_{n}\right]\right)\;$$

(17) $${\bar{h}}_{n}=\;\tan h\;\left(W\cdot \left[r*h_{n-1},\;x_{n}\right]\right)\;$$

(18) $$h_{n}=\left(1-z_{n}\right)*h_{n-1}+z_{n}*\bar{h}$$

Equations (15) and (16) illustrate how $r_{n},\;\;z_{n}\;$ reset, and update gates are evaluated. $W_{z}$ is the weight of $z_{n},$ 0 denotes the sigmoid function,$\;W_{r}$ characterizes the weight of $r_{n}$. A larger value of $z_{n}$ denotes that data were retained through the present cell $r_{n}$ and proposes that when the value corresponds to 0, the dataset from the prior cell is eliminated. Equations (17) and (18) demonstrate the estimation of $\;h_{n}$ and $\bar{h}$ final and pending output of GRU-NN. W characterizes the weight of $z_{n}$, $h_{n-1}$ denotes the output from the preceding cell, and  tanh denotes the hyperbolic tangent function. ${\bar{h}}_{n}$ can be obtained by multiplying $h_{n-1}$ of the prior cell using $r_{n}$ and $x_{n}$, multiplying by W and tanh. $h_{n}$ denotes the amount of two vectors.

#### 3.2.2. LSTM Model

**Figure 2:**
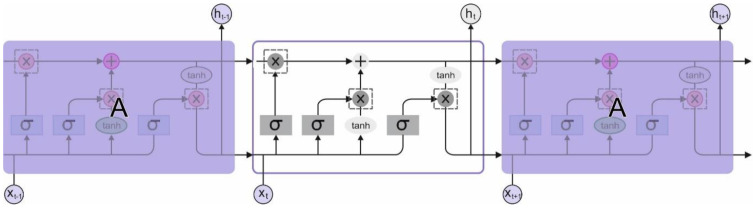
The architecture of LSTM.

The RNN approach was widely employed for predicting and analyzing time sequence datasets. RNN often undergoes the gradient vanishing problem. Hence, it is hard to remember the previous dataset, namely the long dependence problem. To overcome these problems, the LSTM is introduced and applies a gate-controlling method for altering data flow and systematically determines the count of received datasets that are regathered from each time step. Figure 2 represents the architecture of LSTM.

The architecture of the LSTM unit is encompassed by storing unit and three control gates (forget, input, and output gates). $x_{z}$ and $h_{z}$ correspond to the input and hidden state of time z. $f_{z},$ $i_{z}$, and $o_{z}$ determine the forgetting, input, and output gates. ${\vec{C}}_{z}$ indicates the candidate dataset to the input.

(19) $$f_{z}=\sigma ƒ\left(W_{f}\cdot \left[h_{z-1},\;x_{z}\right]+b_{f}\right)$$

(20) $$i_{z}=\sigma \left(W_{i}\cdot \left[h_{z-1},\;x_{z}\right]+b_{i}\right)$$

(21) $$o_{z}=\sigma \left(W_{o}\cdot \left[h_{z-1},\;x_{z}\right]+b_{o}\right)$$

(22) $$\tilde{C}=\;\tan h\;\left(W_{C}\cdot \left[h_{z-1},\;x_{z}\right]+b_{C}\right)$$

(23) $$C_{z}=f_{z}\cdot C_{z-1}+i_{t}\cdot \tilde{C}$$

(24) $$h_{z}=o_{z}\cdot \tan h\;\left(C_{z}\right)$$

$W_{f},$$W_{i},$$W_{o}$, and $W_{c}$$b_{f},$$b_{i},$$b_{o}$, and $b_{c}$ correspondingly denote the weight matrices and bias vector of forget, input, output, and update state. $x^{z}$ represents the time sequence dataset of the existing time interval z, and $h_{z-1}$ denotes the resultant memory unit from the previous time interval z−1.

## 4. Results and Discussion

**Figure 3:**
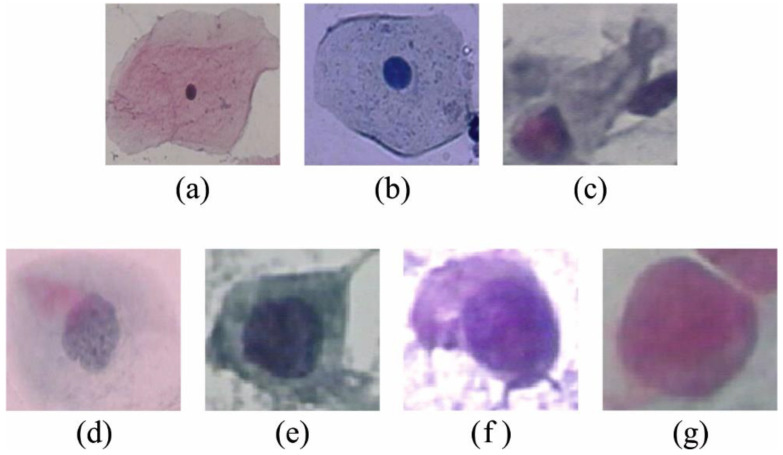
Sample images. (**a**) Superficial squamous (SSE), (**b**) intermediate squamous (ISE), (**c**) columnar (CE), (**d**) mild dysplasia (MS-NKD), (**e**) moderate dysplasia (MOS-NKD), (**f**) severe dysplasia (SS-NKD), (**g**) carcinoma in situ (SCCSI).

The proposed method is simulated using a Python tool. The experimental results of the EOEL-PCLCCI model are tested using the Herlev database [21]. Figure 3 demonstrates some sample images. The proposed model is simulated using Python 3.6.5 tool on PC i5-8600k, GeForce 1050Ti 4 GB, 16 GB RAM, 250 GB SSD, and 1 TB HDD. The parameter settings are learning rate: 0.01, dropout: 0.5, batch size: 5, epoch count: 50, and activation: ReLU.

**Figure 4:**
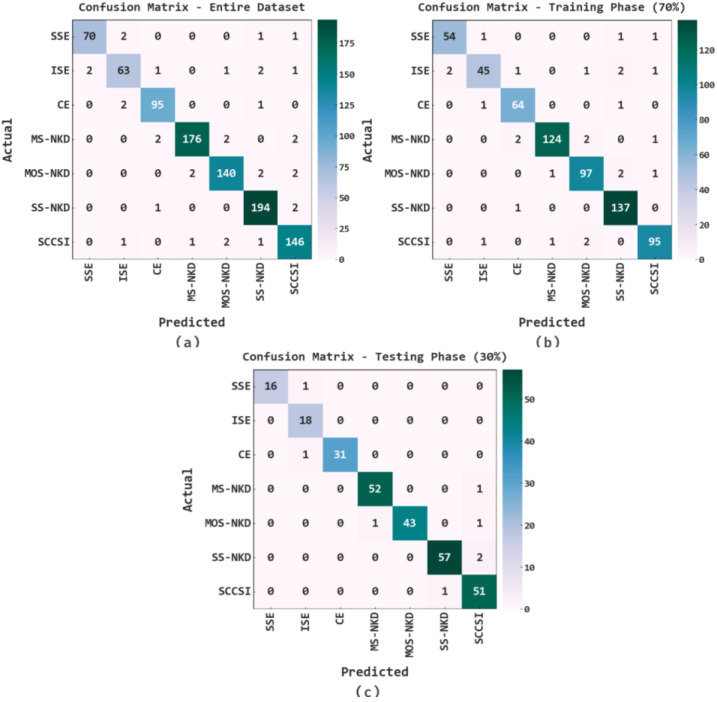
Confusion matrices of EOEL-PCLCCI system on cervical cancer classification; (**a**) entire database, (**b**) 70% of TR database, and (**c**) 30% of TS database.

In Figure 4, the confusion matrices of the EOEL-PCLCCI model on cervical cancer classification performance are provided. The figure implied that the EOEL-PCLCCI model detected all cervical cancer classes.

**Figure 5:**
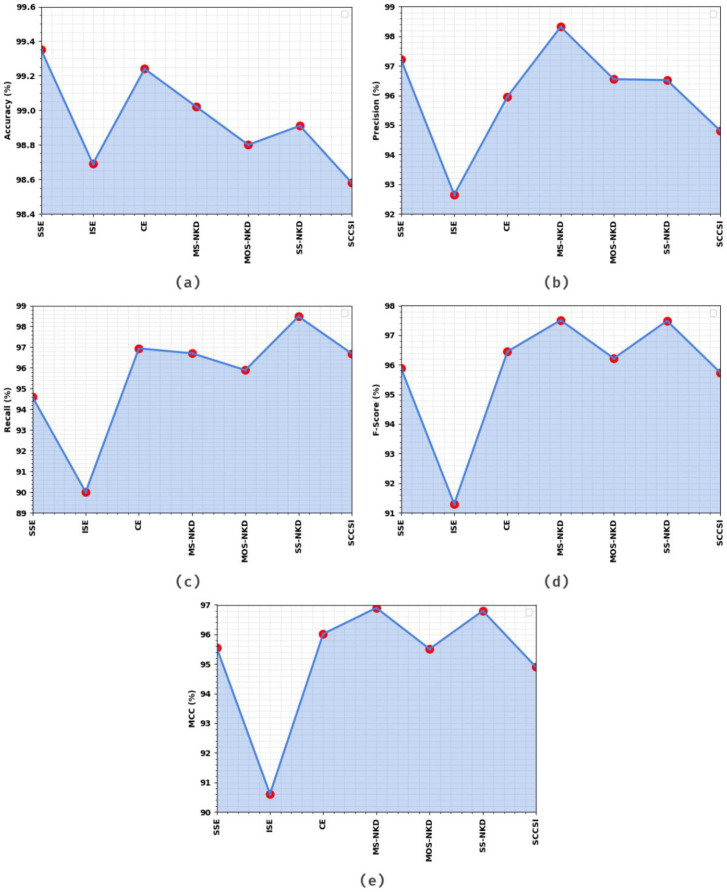
Result analysis of the EOEL-PCLCCI system on the entire database in terms of different measures (**a**) $Accu_{y}$, (**b**) $Prec_{n}$, (**c**) $Reca_{l}$, (**d**) $F_{score}$, and (**e**) MCC.

Table 1 and Figure 5 demonstrate the overall cervical cancer classification results of the EOEL-PCLCCI technique on entire datasets. The experimental value indicates that the EOEL-PCLCCI method has recognized all different class labels. It is observed that the EOEL-PCLCCI approach has reached an average $accu_{y}$ of 98.94%, $prec_{n}$ of 96%, $reca_{l}$ of 95.61%, $F_{score}$ of 95.80%, and MCC of 95.18%.

**Figure 6:**
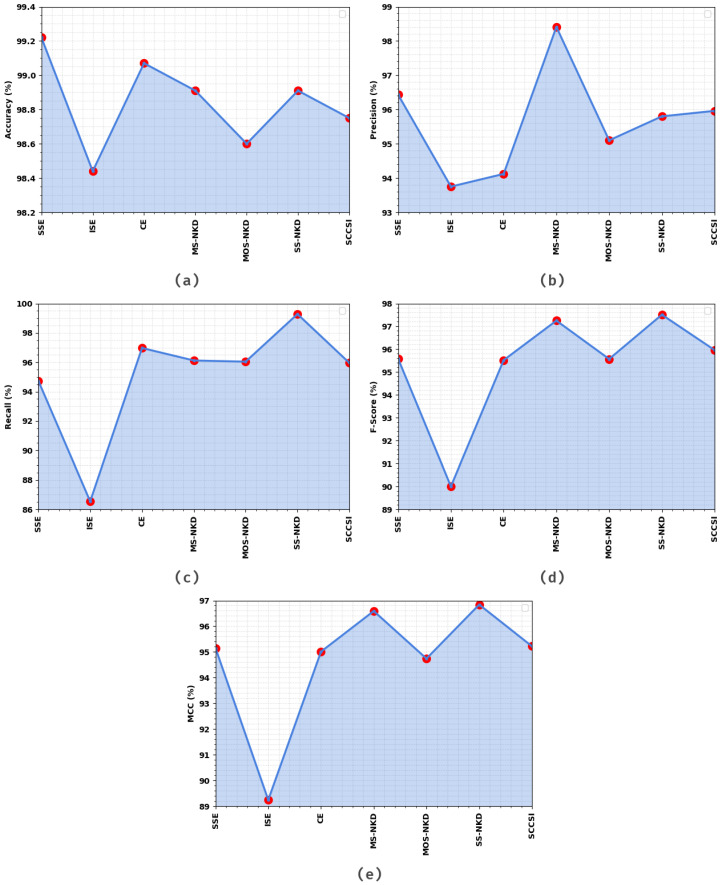
Result analysis of EOEL-PCLCCI system on 70% of TR database in terms of different measures (**a**) $Accu_{y}$, (**b**) $Prec_{n}$, (**c**) $Reca_{l}$, (**d**) $F_{score}$, and (**e**) MCC.

Table 2 and Figure 6 illustrate the overall cervical cancer classification results of the EOEL-PCLCCI technique on the TR database. The simulation values exhibited that the EOEL-PCLCCI approach recognized all different class labels. The EOEL-PCLCCI algorithm has attained an average $accu_{y}$ of 98.84%, $prec_{n}$ of 95.65%, $reca_{l}$ of 95.09%, $F_{score}$ of 95.34%, and MCC of 94.68%.

**Figure 7:**
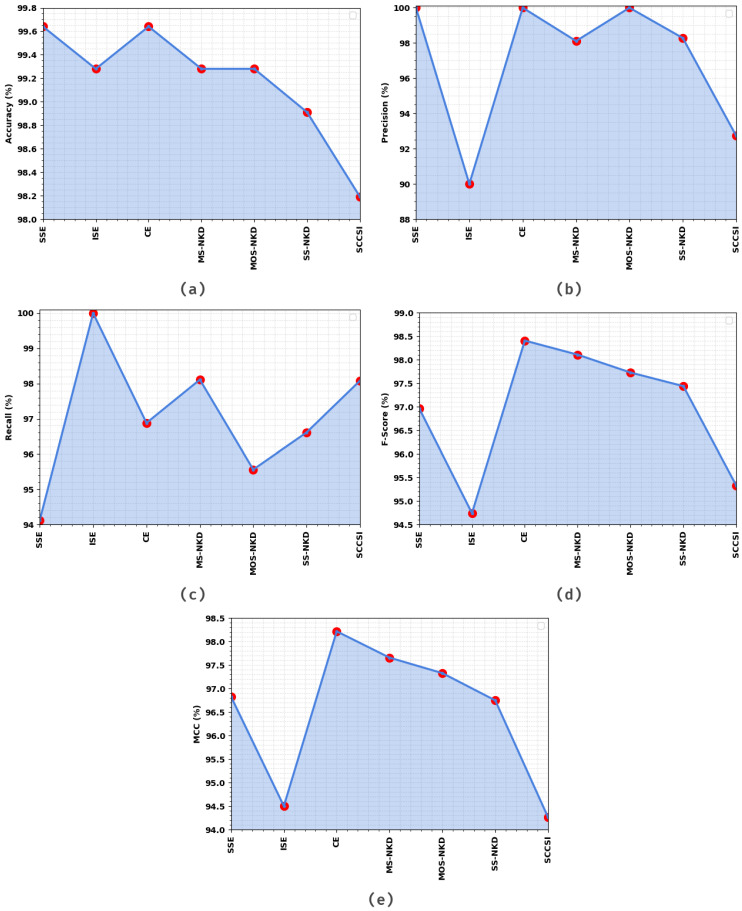
Result analysis of EOEL-PCLCCI system on 30% of TS database in terms of different measures (**a**) $Accu_{y}$, (**b**) $Prec_{n}$, (**c**) $Reca_{l}$, (**d**) $F_{score}$, and (**e**) MCC.

Table 3 and Figure 7 show the overall cervical cancer classification results of the EOEL-PCLCCI approach on the TS database. The simulation values designated that the EOEL-PCLCCI approach has recognized all different class labels. The EOEL-PCLCCI technique has gained an average $accu_{y}$ of 99.17%, $prec_{n}$ of 97.02%, $reca_{l}$ of 97.05%, $F_{score}$ of 96.96%, and MCC of 96.51%.

**Figure 8:**
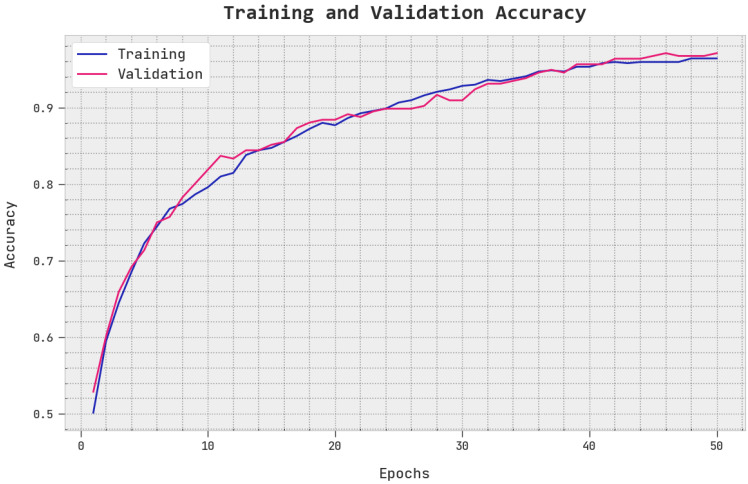
TACC and VACC analysis of EOEL-PCLCCI system.

The TACC and VACC of the EOEL-PCLCCI method are investigated on CC performance in Figure 8. The figure implied that the EOEL-PCLCCI methodology has exhibited improved performance with increased values of TACC and VACC. It is noted that the EOEL-PCLCCI approach has reached maximum TACC outcomes.

**Figure 9:**
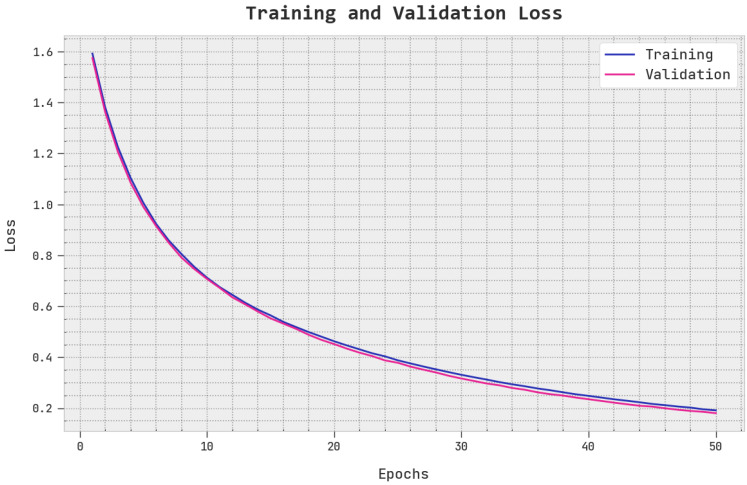
TLS and VLS analysis of EOEL-PCLCCI system.

The TLS and VLS of the EOEL-PCLCCI method are tested on CC performance in Figure 9. The figure designated the EOEL-PCLCCI approach has revealed better performance with minimal values of TLS and VLS. It is noted the EOEL-PCLCCI approach has resulted in reduced VLS outcomes.

**Figure 10:**
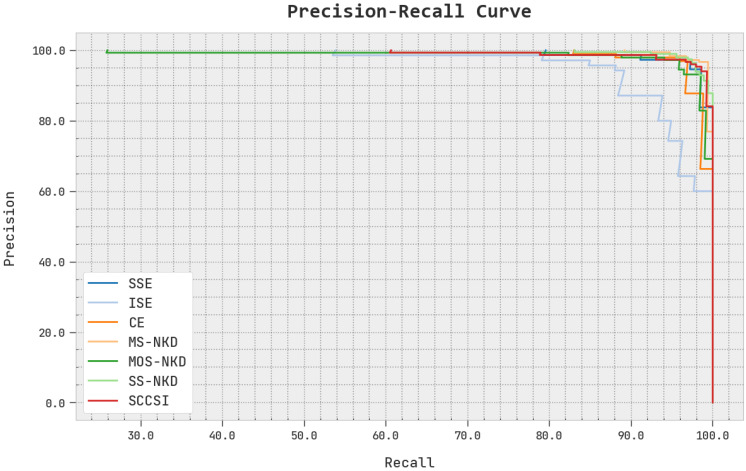
Precision-recall analysis of EOEL-PCLCCI system.

A clear precision-recall inspection of the EOEL-PCLCCI system under test database is shown in Figure 10. The precision-recall curve shows the tradeoff between precision and recall for different threshold. A high area under the curve represents both high recall and high precision, where high precision relates to a low false positive rate, and high recall relates to a low false negative rate. The figure shows the EOEL-PCLCCI method has resulted in superior values of precision-recall value in all the class labels.

**Figure 11:**
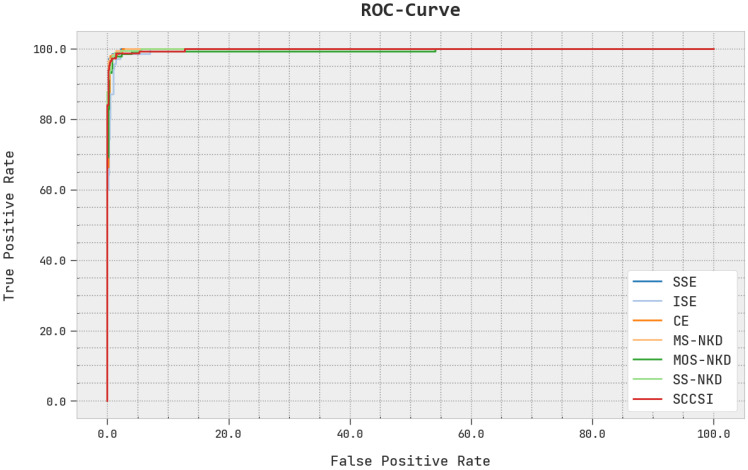
ROC curve analysis of EOEL-PCLCCI system.

The detailed ROC analysis of the EOEL-PCLCCI system under the test database is shown in Figure 11. ROC curves summarize the trade-off between the true positive rate and false positive rate for a predictive model using different probability thresholds. The outcomes exhibited by the EOEL-PCLCCI methodology has signified its ability to categorize distinct classes in test database.

**Figure 12:**
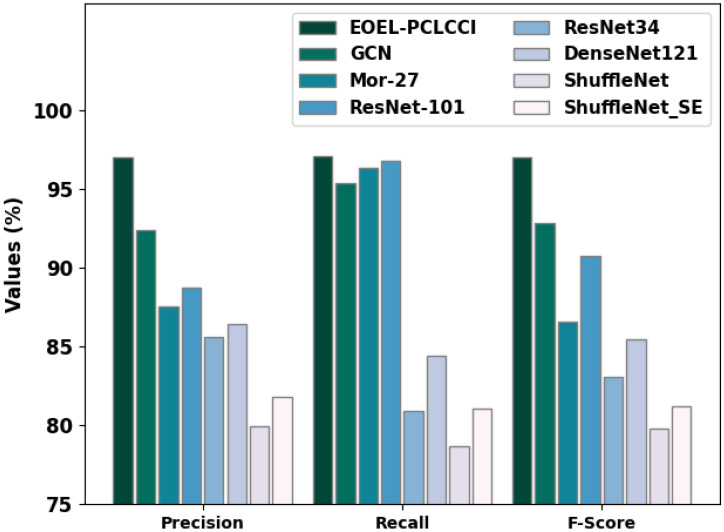
Comparative analysis of EOEL-PCLCCI algorithm with recent approaches.

The experimental results of the EOEL-PCLCCI model are compared with other DL models in Table 4 and Figure 12 [24,25]. The result implies that the ShuffleNet and ShuffleNet_SE models have shown lower performance, whereas the ResNet34 and DenseNet121 models have reported moderately improved performance.

In contrast, the Mor-27 and ResNet-101 models have tried to obtain reasonable outcomes. Although the GCN model has shown near-optimal performance, the EOEL-PCLCCI model has shown enhanced results with $accu_{y}$ of 99.17%, $prec_{n}$ of 97.02%, $reca_{l}$ of 97.05%, and $F_{score}$ of 96.96%. Therefore, the EOEL-PCLCCI model has shown superior results over other models.

## 5. Conclusions

In this study, we have introduced an automated cervical cancer classification method, named EOEL-PCLCCI algorithm on colposcopy images. In the presented EOEL-PCLCCI technique, the DenseNet-264 architecture is used for feature extraction and EO algorithm is applied as a hyperparameter optimizer. For classification process, an ensemble of weighted voting classifiers namely GRU and LSTM is used. A widespread simulation analysis is performed on benchmark dataset to depict the superior performance of the EOEL-PCLCCI technique, and the results demonstrate the superiority of the EOEL-PCLCCI algorithm over other DL models with maximum accuracy of 99.17%. Thus, the EOEL-PCLCCI approach can be used for cervical cancer classification effectively. In the future, the performance of EOEL-PCLCCI technique needs to be enhanced by deep instance segmentation.

## Figures and Tables

**Table 1:** CC outcome of EOEL-PCLCCI system with various classes under entire database.

Entire Dataset					
Labels	$Accu_{y}$	$Prec_{n}$	$Reca_{l}$	$F_{score}$	MCC
SSE	99.35	97.22	94.59	95.89	95.55
ISE	98.69	92.65	90.00	91.30	90.61
CE	99.24	95.96	96.94	96.45	96.02
MS-NKD	99.02	98.32	96.70	97.51	96.90
MOS-NKD	98.80	96.55	95.89	96.22	95.51
SS-NKD	98.91	96.52	98.48	97.49	96.80
SCCSI	98.58	94.81	96.69	95.74	94.90
Average	98.94	96.00	95.61	95.80	95.18

**Table 2:** CC outcome of EOEL-PCLCCI system with various classes under TR database.

Training Phase (70%)					
Labels	$Accu_{y}$	$Prec_{n}$	$Reca_{l}$	$F_{score}$	MCC
SSE	99.22	96.43	94.74	95.58	95.15
ISE	98.44	93.75	86.54	90.00	89.24
CE	99.07	94.12	96.97	95.52	95.01
MS-NKD	98.91	98.41	96.12	97.25	96.59
MOS-NKD	98.60	95.10	96.04	95.57	94.74
SS-NKD	98.91	95.80	99.28	97.51	96.84
SCCSI	98.75	95.96	95.96	95.96	95.22
Average	98.84	95.65	95.09	95.34	94.68

**Table 3:** CC outcome of EOEL-PCLCCI system with various classes under TS database.

Testing Phase (30%)					
Labels	$Accu_{y}$	$Prec_{n}$	$Reca_{l}$	$F_{score}$	MCC
SSE	99.64	100.00	94.12	96.97	96.83
ISE	99.28	90.00	100.00	94.74	94.50
CE	99.64	100.00	96.88	98.41	98.22
MS-NKD	99.28	98.11	98.11	98.11	97.66
MOS-NKD	99.28	100.00	95.56	97.73	97.33
SS-NKD	98.91	98.28	96.61	97.44	96.75
SCCSI	98.19	92.73	98.08	95.33	94.26
Average	99.17	97.02	97.05	96.96	96.51

**Table 4:** Comparative analysis of EOEL-PCLCCI algorithm with recent approaches.

Methods	$Accu_{y}$	$Prec_{n}$	$Reca_{l}$	$F_{score}$
EOEL-PCLCCI	99.17	97.02	97.05	96.96
GCN	96.28	92.41	95.38	92.79
Mor-27	94.34	87.55	96.36	86.57
ResNet-101	91.58	88.70	96.75	90.73
ResNet34	83.47	85.59	80.94	83.08
DenseNet121	86.40	86.45	84.42	85.46
ShuffleNet	79.78	79.97	78.66	79.78
ShuffleNet_SE	80.90	81.79	81.04	81.22

## Data Availability

Data sharing does not apply to this article as no datasets were generated during the current study.
